# Chemical investigation and anti-trichinellosis activity of some ficus species leaves: in silico supported in vitro study

**DOI:** 10.1186/s12906-026-05500-5

**Published:** 2026-08-14

**Authors:** El-Sayed S. Abdel-Hameed, Ezzat E. A. Osman, Heba R. Mohamed, Mortada M. El-Sayed, Hend Okasha, Hagar F. Abdelmaksoud, Eman S. El-Wakil

**Affiliations:** 1 Department of Medicinal Chemistry, Theodor Bilharz Research Institute, Giza, 12411 Egypt; 2 Biochemistry and Molecular Biology Department, Theodor Bilharz Research Institute, Giza, 12411 Egypt; 3 Department of Parasitology, Theodor Bilharz Research Institute, Kornaish El-Nile St, Giza, 12411 Egypt

**Keywords:** *Ficus*, *Trichinella spiralis*, In vitro analysis, Electron microscopy, GC-MS, In silico study

## Abstract

**Background:**

Worldwide, trichinellosis is a zoonotic illness. Albendazole (ALB), the nowadays anti-trichinellosis drug, has side effects and insufficient effectiveness. *Ficus* species have ubiquitous health benefits. The current work aimed to assess the in vitro anti-trichinellosis potential of seven *Ficus* species compared to the reference drug, ALB.

**Method:**

Fourteen crude extracts from seven *Ficus* species (spp.) leaves were prepared using 70% methanol and aqueous solvents. *Trichinella spiralis* (*T. spiralis*) adult worms were isolated from infected mice, cultivated on a 24-well tissue culture plate, and subjected to the crude *Ficus* spp. extracts and ALB in culture media. The efficacy of the tested compounds was evaluated using parasitological analysis and electron microscopy (SEM). Preliminary phytochemical screening and total phenolic content were performed on *Ficus* spp. extracts showing the most promising effects. In addition, GC-MS analysis was performed on the most active extract to determine its chemical composition.

**Results:**

The parasitological analysis revealed that *Ficus microcarpa* (*F.m*) had the most promising effects, followed by *Ficus sycomorus* (*F.s*) and *Ficus nitida* (*F.n*) with the methanolic extracts’ LC_50_ of 12.8 µg/mL, 31.5 µg/mL, and 42.9 µg/mL, respectively, compared to the ALB (52.5 µg/mL). SEM results documented changes and a loss of the typical tegumental structure and morphology in adult *T. spiralis* worms. The GC-MS analysis of *F.m* methanolic extract yielded 26 compounds, including fatty acids, esters, tricyclic diol, and glucosinolates. Molecular docking studies further supported these results, with high binding affinities among the major compounds of *F.m* and the tubulin beta chain of *T. spiralis*, an essential protein for parasite survival.

**Conclusion:**

The current study emphasizes the promising potential of *Ficus* species, especially *F.m*, as natural sources for the development of antiparasitic agents for the therapy of trichinellosis.

**Supplementary Information:**

The online version contains supplementary material available at 10.1186/s12906-026-05500-5.

## Introduction

Trichinellosis, often known as trichinosis, is a foodborne parasite infection caused by roundworms (nematodes) belonging to the genus *Trichinell*a [1]. *Trichinella spiralis* (*T. spiralis*) is brought on by eating raw or undercooked meat, typically pork. It can cause symptoms that range from mild ones like generalized fever, abdominal discomfort, diarrhea, nausea, vomiting, and myalgia to more serious ones like myocarditis and encephalitis [2]. Around 10,000 cases of trichinellosis are thought to occur annually, and 11 million people around the world [3]. Antiparasitic medications, including Albendazole (ALB) and mebendazole, are used to treat infections caused by *T. spirali*s. Despite their broad activity, moderate toxicity, and high therapeutic index, their efficacy is limited by poor water solubility, low absorption, and weak activity against encysted larvae. Furthermore, several of these medications are not suitable for pregnant women or children under the age of two [4].

Given the significant burden of this parasitic disease and the limited treatment options, finding novel medications to manage and cure *T. spiralis* infection is urgently needed.

Since ancient times, natural materials have been used to treat human diseases; consequently, many contemporary medications are derived from natural molecules [5]. Researchers are still working hard to find novel, physiologically active natural compounds. Plants have long been recognized as a valuable source of chemicals with medicinal potential, and many of the main medications on the market today are made from natural ingredients [6].

One of the most varied genera in the Moraceae family is Ficus, which includes over 800 species in tropical and subtropical climates and is commonly known as fig trees [7]. About 100 *Ficus* species are growing in Africa. Additionally, several categories of substances, including flavonoids, phenolics, terpenoids, tannins, alkaloids, lactones, amino acids, lignans, and coumarins, were identified as a result of phytochemical research conducted on the *Ficus* species [8]. Most documented health benefits of *Ficus* species have been linked to antioxidant, antimicrobial, antidiabetic, anticancer, antibacterial, and antirheumatic properties [9].

The ethnopharmacological significance of the genus *Ficus* as a medicinal resource with widespread traditional use for infectious, gastrointestinal, and worm-related conditions was the primary factor in selecting the seven *Ficus* species. Since many *Ficus* species are commonly used for symptoms such as diarrhea, dysentery, intestinal problems, and other infection-associated conditions that overlap with parasitic disease presentations, this genus-level rationale is relevant for initial screening research [10]. Furthermore, a variety of secondary metabolites, including phenolics, flavonoids, tannins, and triterpenoids, are known to be present in the *Ficus* genus. Ficin, a proteolytic enzyme, is found in high concentrations in the latex of the genus *Ficus*. These compounds are generally thought to have antiparasitic properties. Documented anthelmintic and antiprotozoal effects of certain *Ficus* species support this rationale [11].

Therefore, this study aims to evaluate the potential anti-*T. spiralis* effects of extracts from seven *Ficus* species compared to the reference drug, ALB. Then, the extracts with promising activities will be subjected to phytochemical screening, and GC-MS analysis will be performed on the most active extract to identify its chemical composition.

## Materials and methods

### Plant material

The leaves of seven *Ficus* species: *Ficus benjamina* (*F. b*), *Ficus carica* (*F. c*), *Ficus elastica* (*F. e*), *Ficus microcarpa* (*F. m*), *Ficus nitida* (*F. n*), *Ficus sycomorus* (*F. s*), and *Ficus virnes* (*F. v*) were collected from Al-Sharkia and Giza Governorates, Egypt. Mrs. Teraza Labib, a taxonomy specialist at Orman Herbarium Garden, authenticated the plant samples. The voucher specimens (No. 20220301 for *F.b*; No. 20220302 for *F.c*; No. 20220303 for *F. e*; No. 20220304 for *F. m*; No. 20220305 for *F.n*; No. 20220306 for *F.s*; No. 20220307 for *F.v*) were deposited at the herbarium of the Medicinal Chemistry Department, Theodor Bilharz Research Institute (TBRI). The leaves were then dried at an ambient temperature in a shaded place, ground into a fine powder, and kept in the dark till extraction.

### Extraction process

Dry powdered leaves of the seven *Ficus* species (500 g) were soaked in two different solvent systems, which are 70% MeOH (ME) and distilled water (AE), affording two different extracts of each plant species. In the current study, 70% MeOH was used rather than absolute MeOH because hydroalcoholic mixtures are widely recognized as optimal for extracting a broad spectrum of plant metabolites, particularly phenolic and other polar-to-moderately polar compounds. The presence of water in the solvent system enhances swelling of plant tissues and the solubilization of more polar constituents, leading to higher total phenolic yields and stronger bioactivity than absolute methanol. Several studies have reported that 60–80% aqueous methanol or ethanol provides superior extraction efficiency for phenolics and related phytochemicals [12]. The crude extracts were then filtered and concentrated using a rotary evaporator (BUCHI, Germany) under reduced pressure and temperature at 45 °C. This procedure has been carried out three times for each extract. The AE extracts were subsequently dried by lyophilization [13]. The fourteen crude extracts were kept in dark, clean vials for further analysis [14].

### Ethics approval

The current study was carried out with approval from the Research Ethical Committee (REC) of the TBRI, under protocol number PT (697). TBRI-REC was operated in accordance with the National Institutes of Health (NIH) guidelines for the care and use of laboratory animals (eighth edition) and adhered to the ARRIVE guidelines.

### Isolation of *T. spiralis* adult worms

Adult *T. spiralis* worms were intentionally selected for the present study based on the following clinical rationale. When the adult worms establish in the host intestine, they produce newborn larvae, which then migrate and encyst in host skeletal muscles, resulting in severe pathology, myositis, and metabolic complications of trichinellosis. So, interference at this stage may effectively prevent further progression of infection and interrupt the parasite’s life cycle at the enteral phase, directly halting fecundity and stopping muscle-stage pathogenesis before it can initiate [15]. In contrast, muscle larvae are more relevant to studies of chronic infection than to the initial intestinal phase. Additionally, enzymatic digestion of muscle larvae has been shown to induce cuticular artifacts that can confound morphological assessments [16].

In the present work, *T. spiralis* adults (code: ISS6158) were obtained from infected laboratory-bred Swiss albino mice (male, age ranges from 5 to 7 weeks, weight ranges from 20 to 25 g and housed in polypropylene cages with unrestricted access to food and water under regulated conditions (22–24 °C and a 12-hour dark/light cycle)), courtesy of the parasitology department at TBRI, Giza, Egypt. Pepsin-HCl (1%) solution was used to mince and digest the infected muscles to obtain the muscle larvae required for infection. After overnight incubation at 37 °C, the larvae were collected by sedimentation. They were then rinsed repeatedly using saline, and their number per mL was counted [17].

Mice were infected orally with 200–300 *T. spiralis* larvae [18].

Throughout the study, the infected mice received regular commercial pelleted food and water as needed and were kept hygienically. Five days after infection (dpi), adult worms were collected. Infected mice (*n* = 20) were euthanized by isoflurane (Forane^®^, UK) as the sole anesthetic agent at a concentration of 3–5% via inhalation, resulting in rapid loss of consciousness. Following the NIH Guidelines for the Care and Use of Laboratory Animals (2011) [19] and the AVMA for Animals Euthanasia (2020) [20], euthanasia was carried out under deep anesthesia.

After that, the infected mice’s intestines were cut out with scissors. The intestines were gently cleaned in phosphate-buffered saline (PBS) to remove intestinal contents. The small intestines were then divided into 2 cm pieces lengthwise and placed on gauze in a beaker containing 250 mL of PBS for three hours at 37 °C [21].

### In vitro experimental design

On a 24-well tissue culture plate, adult worms of *T. spiralis* (25 parasites per well) were cultured using RPMI-1640 media that contained 200 U/mL penicillin, 200 µg/mL streptomycin, and 20% fetal bovine serum. The ALB concentration range and the *Ficus* extracts against adults were 25–400 µg/mL [22]. Dimethyl sulfoxide (DMSO) at 0.5-1% and parasite controls were established. Three independent biological experiments were performed, and worms were equally allocated to treatment wells in each run. The plates were incubated for 1, 4, 24, 48, and 72 h at 37 °C with 5% CO2.

### Parasitological analysis

Inverted microscopy ( ZEISS, USA) was used to count the dead and living parasites in the wells after the incubation periods. The adult motility assay is the preferred technique for assessing anthelmintic susceptibility across various worm species, and a blinded investigator carried out the mortality assessment. The survival rate was calculated in each well, and non-motile worms were regarded as dead [23].

### Scanning electron microscopy (SEM)

Adult worms were incubated with the compounds that showed the most promising effects in the in vitro assay at concentrations equal to their corresponding LC_50_ for 48 h. 10 worms per group were examined in each independent experimental replicate, and representative images were taken in a blinded manner. The adult worms were treated using the techniques described by Abou Rayia et al. [18]. Worms were promptly fixed in a new fixation solution (glutaraldehyde solution (2.5%) at pH 7.2 buffered with 0.1 M sodium cacodylate) and stored at 4 °C overnight. The fixed specimens were then rinsed in distilled water, post-fixed in 2% osmium tetroxide for one hour, and washed in 0.1 M sodium cacodylate buffer at pH 7.2 for five minutes. After being dehydrated in a graded ethyl alcohol series (50%, 70%, 80%, 90%, 95%, and two changes of 100%), the samples were placed on carbon-coated adhesive material, and the FEI-Philips Quanta 250 scanning electron microscope (Model No. 1027641; FEI Company, Czech Republic) was used for examination [24, 25].

### Preliminary phytochemical screening

Preliminary phytochemical screening of the bioactive ingredients (flavonoids, alkaloids, tannins, phenols, and saponins) was performed on the ME and AE extracts, which had the most promising anti-*T. spiralis* effects in the in vitro studies following the standard analysis procedures [26].

### Total phenolic content (TPC)

The TPC was determined using the Folin-Ciocalteu procedure [27]. Briefly, Folin-Ciocalteu reagent (2.5 mL) was combined with 0.5 mL of the extract/s (200 µg/mL), then 2 mL of Na_2_CO_3_ (7.5%, w/v) was added. The reaction was incubated in the dark at room temperature for 30 min. The absorbance was measured at 760 nm (UV–Vis spectrophotometer, Milton Roy Spectronic 601, USA), with a reagent blank (containing the same concentration of DMSO) used for baseline correction. The data are presented as the mean ± SD (*n* = 3) of mg of gallic acid equivalents (GAE) per gram of dry extract.

### Gas chromatography/mass spectrometry (GC-MS) analysis

The most active *Ficus* extract in the in vitro studies was diluted in an appropriate volume of methanol, and 1 µL of the sample was injected into a GC-TSQ mass spectrometer (Thermo Scientific, USA) with a direct capillary column TG–5MS (30 m x 0.25 mm x 0.25 μm film thickness). The column oven temperature was first adjusted to 60 °C, then raised to 250 °C by 5 °C/min with a 2-minute hold, and finally raised by 30 °C/min to 300 °C. The injector temperature was held at 270 °C. Helium was used as the carrier gas at a steady flow rate of 1 mL/min. The diluted sample was automatically injected by the AS3000 autosampler paired with the GC in split mode after a 4-minute solvent delay. EI mass spectra were collected in full scan mode using 70 eV ionization voltages across the *m/z* range of 50–650 Da. The ion source and transfer line temperatures were adjusted to 200 °C and 280 °C, respectively. Metabolites were identified by comparing their mass spectra with those from the WILEY 09 and NIST 14 spectral databases.

### Molecular docking study

In this study, the most abundant compounds identified in the most active *Ficus* extract were subjected to in silico molecular docking analysis to evaluate their potential binding affinity to the β-tubulin protein of *T. spiralis*. The amino acid sequence of the tubulin beta chain for *T. spiralis* (Accession Number: E5SWF2) was retrieved from the UniProtKB database (uniprot.org/). Due to potential constraints imposed by predefined structures, template-based homology modeling was used to generate 3D models of the target protein, the tubulin beta chain.

For this purpose, a web-based server for protein structure (SWISS-MODEL (swissmodel.expasy.org/)) was employed. This service searches the Protein Data Bank (PDB) and the SWISS-MODEL Template Library (SMTL) for appropriate template structures for the target protein using a combination of BLASTp and HHBlits.

The identified templates were then utilized to generate a high-confidence structural model of the target protein sequence. The Z-scoring functions, General Model Quality Estimate (GMQE), and Qualitative Model Energy Analysis (QMEAN), which are specific to SWISS-MODEL outputs, were used to assess the quality of the generated models [28–30]. These scores objectively assess the model’s accuracy and reliability. The 3D tubulin beta chain protein model, co-crystallized with the colchicine ligand, was downloaded in PDB format and prepared as a docking receptor.

For the prediction of both the affinity of protein-ligand binding and the preferred docking pose orientation between the amino acid residues that constitute the tubulin beta chain colchicine binding pocket and the studied compounds, in addition to the co-crystallized ligand (colchicine) and ALB, that were used as reference ligands, AutoDock Vina modeling simulation software (AutoDock Vina v.1.2.0) was used.

The tubulin beta chain was extracted from its co-crystalized colchicine using the PyMOL molecular visualization tool (PyMOL v.2.5.4) (Schrödinger, Inc.) after hydrogen atoms were added to both with the extracted files in PDB format [31].

The ligand binding pocket’s docking site and grid box dimensions were determined using Auto-Dock (MGL-tools) [32]. The grid box was centered on the colchicine ligand in the crystal structure, with the grid center coordinates set to X = 15.867, Y = 65.349, Z = 42.643 Å, a grid box dimension of 26 × 24 × 36 Å, and a spacing of 1.0 Å, as described in Table 1. Furthermore, Open Babel v.2.3.1 exported the target protein and tested compounds in PDBQT format (AutoDock) [33].

**Table 1:** Grid box dimensions of colchicine binding pocket

Parmeter	Value
Grid center (x; y; z)	15.867; 65.349; 42.643 Å
Grid box dimensions (x; y; z)	26; 24; 36 Å
Spacing	1.0 Å

The tested compounds’ two-dimensional (2D) structures were retrieved from the PubChem database (pubchem.ncbi.nlm.nih.gov). Subsequently, Open Babel v.2.3.1 was used to construct 3D structures, assign protonation states at physiological pH 7.4, calculate partial charges, and perform shape optimization and energy reduction using the MMFF94 force field to improve the docking simulation’s accuracy. Moreover, Open Babel exported the target proteins and the tested compounds in PDBQT (AutoDock) format.

The target protein was a rigid receptor, and the ligands’ conformations were flexible [34]. A maximum of 9 poses were scored for each docking run for each molecule. A total of 10 independent docking runs were performed for each ligand to ensure reproducibility and proper conformational sampling. The exhaustiveness parameter was set to 32 to increase the global search of the conformational space. The best-fit pose was defined as the protein-ligand complex with the lowest free energy. It was further validated by visual inspection of the clustering of the top-scoring poses. The final binding pose was selected as the one with the lowest binding energy (ΔG) for each compound. Colchicine was docked again into the modeled colchicine-binding pocket of the *T. spiralis* β-tubulin homology model to validate the docking protocol. The root-mean-square deviation (RMSD) of the docked pose and the reference conformation (co-crystallized colchicine extracted from the template PDB: 4O2B) was calculated using PyMOL. An RMSD value ≤ 2.0 Å was taken as acceptable, confirming the reliability of the docking procedure to reproduce the native binding geometry [34]. Finally, the most suitable pose was chosen based on the protein-ligand complex’s lowest free energy. BIOVIA Discovery Studio (DS) Visualizer v.4.5 was used to examine ligand-protein interactions and visualize binding modes and intermolecular interactions, including hydrogen bonds, hydrophobic contacts, and electrostatic interactions.

### Statistical analysis

The data were presented as the mean ± SD of three independent biological experiments. All statistical analyses were done using GraphPad Prism 10 (California, USA). One-way analysis of variance (ANOVA) was used to compare multiple groups, and Tukey’s post hoc test was used for pairwise comparisons between treatment groups and controls. The corrected worm mortality was calculated relative to the DMSO control. Dose–response curves were fitted using nonlinear regression (log(inhibitor) vs. response—variable slope) to determine the median lethal concentration (LC_50_) values and 95% confidence intervals. A *p*-value < 0.05 was considered a statistically significant difference.

## Results

### Parasitological analysis

**Fig. 1:**
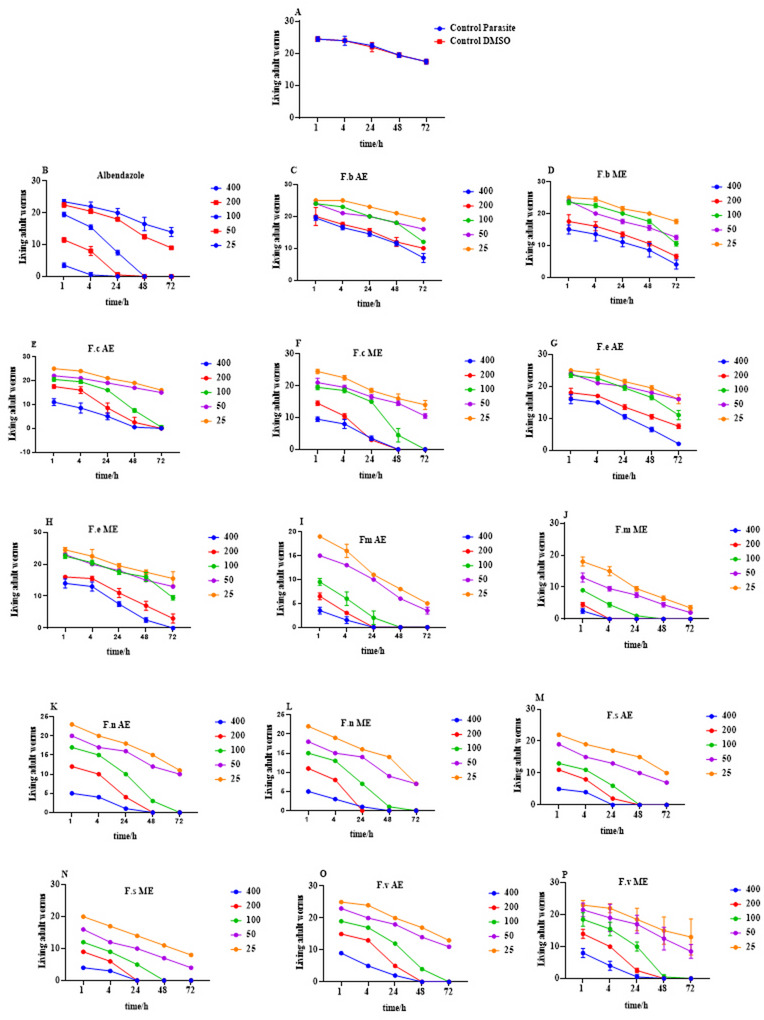
Survival number (Mean ± SD of three independent experiments (*n* = 3)) of *T. spiralis* adults. **A** DMSO and parasite controls, (**B**) ALB, (**C**, **D**) *Ficus benjamina* (*F.b*), (**E**, **F**) *Ficus carica* (*F.c*), (**G**, **H**) *Ficus elastica* (*F.e*), (**I**, **J**) *Ficus microcarpa* (*F.m*), (**K**, **L**) *Ficus nitida* (*F.n*), (**M**, **N**) *Ficus sycomorus* (*F.s*), and (**O**, **P**) *Ficus virnes* (*F. v*). ME: (70% MeOH), AE: (distilled water)

The survival rates of *T. spiralis* adults cultured at varying concentrations of the compounds for different exposure durations, and the LC_50_ values of different *Ficus* species, are shown in Fig. 1; Table 2, respectively. The adult worm survival numbers (Mean ± SD) displayed in Supplementary Table 1 revealed a dose- and time-dependent anthelmintic effect across all tested extracts and the reference drug ALB. ALB exhibited a potent activity, eliminating adult worms at 100 µg/mL within 24 h, with an LC_50_ of 52.5 µg/mL at 48 h. Further analysis of the survival rates revealed that *F.m* ME exhibited the highest anthelmintic activity among all tested plant extracts, achieving complete adult worm mortality at concentrations ≥ 200 µg/mL within 4 h and an LC_50_ of 12.8 µg/mL. The AE of the same species also demonstrated significant efficacy (LC_50_ = 18.9 µg/mL), showing complete worm death at 400 µg/mL by 24 h. Other effective extracts included the ME and AE of *F.s* with LC_50_ values of 31.5 µg/mL and 45.2 µg/mL, respectively. In addition, the ME and AE of *F.n* also showed promising activity (LC_50_ = 42.9 µg/mL and 53.6 µg/mL, respectively), reducing adult worm survival in a dose- and time-dependent manner. Likewise, *F.v* ME achieved substantial worm reduction, with an LC_50_ of 49.8 µg/mL, while the aqueous extract displayed slightly lower potency (LC_50_ = 63.2 µg/mL). All extracts showed statistically significant reductions in worm survival compared to controls (*p* < 0.05), especially at higher concentrations and extended exposure times. The DMSO and parasite controls maintained high survival rates, confirming the validity of the experimental setup.

**Table 2:** LC_50_ of ALB and different *Ficus* species

Drugs	ALB	F.b		F.c		F.e		F.m		F.*n*		F.s		F.v	
		AE	ME	AE	ME	AE	ME	AE	ME	AE	ME	AE	ME	AE	ME
LC_50_ (48 h) µg/mL	52.5	302.2	292	86.7	65.8	213.3	163.4	18.9	12.8	53.6	42.9	45.2	31.5	63.2	49.8

*F.b* Ficus benjamina, *F.c* Ficus carica , *F.e* Ficus elastica, *F.m* Ficus microcarpa, *F.n* Ficus nitida, *F.s* Ficus sycomorus, and *F.v* Ficus virnes

ME (70% MeOH), AE (distilled water)

### Scanning electron microscope analysis

**Fig. 2:**
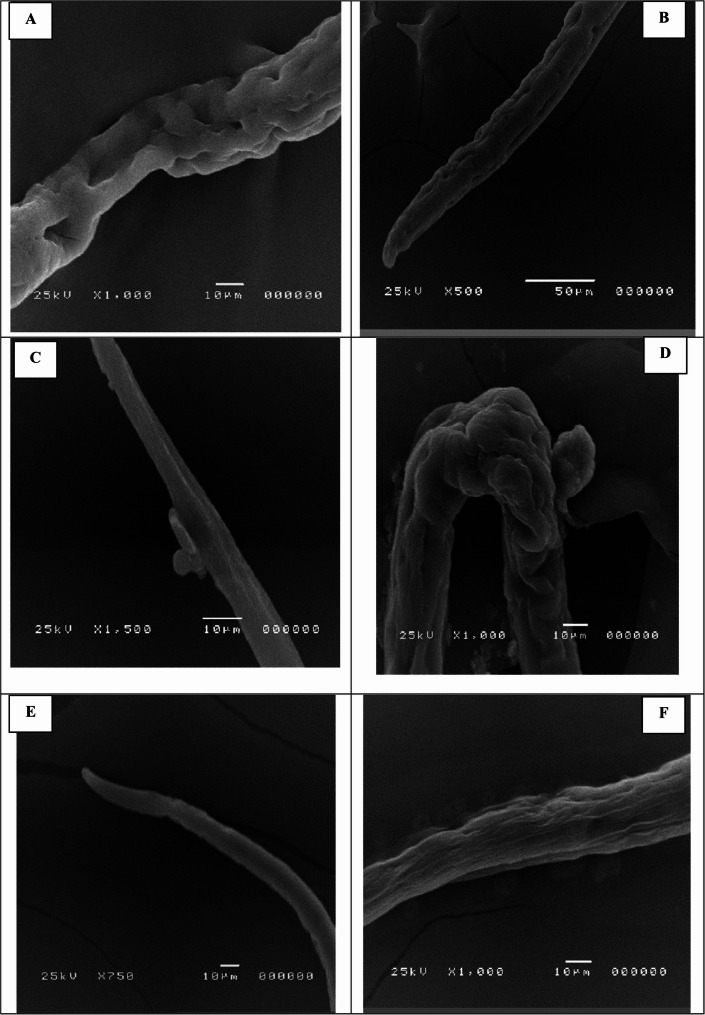
SEM of Adult worms; **A**, **B**: Normal adult worms with typical structure; **C**: Albendazole-treated groups; **D**, **E**, **F**: *F.m*,* F.s*, and *F.n* treated groups, respectively

In the control group, incubated in either DMSO or blank culture media, the adult worm’s cuticle maintained its characteristic structure, including ridges, annulations, transverse creases, and openings of the hypodermal gland (Fig. 2A, B).

Significant adult worm destruction was evident in the ALB-treated group. Cauliflower masses appeared, and the annulations were lost, with the typical creases gone (Fig. 2C).

Regarding different extracts of *Ficus* spp., *F.m* demonstrated the most promising effects, followed by *F.s* and *F.n*. Consequently, SEM analysis was performed on these treated groups. Normal morphology was lost, and the treated groups no longer had the usual creases or annulations, with the appearance of cauliflower masses (Fig. 2D, E, F).

### Preliminary phytochemical screening

According to the results of in vitro anti-*T. spiralis* activity, preliminary phytochemical screening was performed on the AE and ME of three *Ficus* spp., *F.m*,* F.s*, and *F.n*, exhibiting the highest activities. The preliminary phytochemical screening of these crude extracts displayed the existence of several phytochemicals (Table 3). The most abundant phytoconstituents in the ME were flavonoids, phenols, tannins, and saponins, while alkaloids were found in moderate amounts. The AE of these species showed moderate levels of flavonoids, phenols, and tannins, and low levels of alkaloids and tannins.

**Table 3:** Preliminary phytochemical screening of the most active extracts of *Ficus spp.*

Phytochemical	MeOH extract			Aqueous extract		
	*F. m*	*F. n*	*F. s*	*F. m*	*F. n*	*F. s*
Flavonoids	+++	+++	+++	++	++	++
Alkaloids	++	+	++	+	–	+
Tannins	++	++	+++	+	+	++
Phenols	+++	+++	+++	++	++	+
Saponins	+++	++	++	++	+	++

*F.m* Ficus microcarpa, *F.n* Ficus nitida, *F.s* Ficus sycomorus

(+++): high, (++): moderate, (+): low, (-): absent

### Total phenolic content

The ME of *F.n* leaves possessed more phenolics than the other crude extracts (Table 4), followed by the ME of *F.m* and then that of *F.s*. The aqueous extracts of the tested samples had lower phenolic content than their methanolic extracts.

**Table 4:** Total phenolic content of the most active extracts of *Ficus spp.*

	MeOH extract			Aqueous extract		
	*F.m*	*F.n*	*F s*	*F m*	*F.n*	*F.s*
TPC (mg GAE/ g extract)	79.86±0.84	265.23±0.84	62.27±0.84	76.20±2.82	198.19±1.93	76.20±1.93

*F. m* Ficus microcarpa, *F.n* Ficus nitida, *F.s* Ficus sycomorus, *TPC* total phenolic content, *GAE* gallic acid equivalents

The results are displayed as Mean ± SD (*n* = 3)

### Gas chromatography/mass spectrometry analysis

**Fig. 3:**
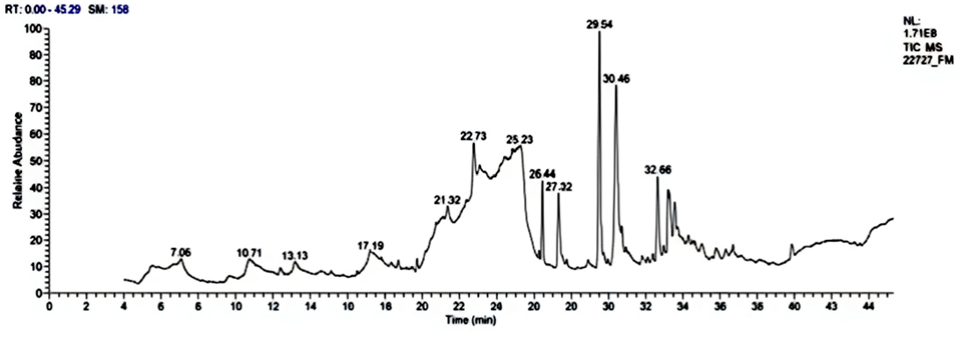
Total-ion GC–MS chromatogram of the ME of *F. m*

Results in Fig. 3 demonstrate the total ion chromatogram of the ME of *F. m.*, which possessed the highest antiparasitic potential. Twenty-six compounds, constituting 88.32% of its total composition, were separated, and 24 were identified, representing 83.34% of its overall composition. Table 5 lists these compounds’ names, molecular formulas, molecular weights, peak area percentages, and retention times. Table 6 presents the major metabolites in the extract along with their chemical nature, hit spectra, chemical structures, and documented bioactivity, if any. In addition to 9,12,15-octadecatrienoic acid, methyl ester, (Z, Z, Z)- (linolenic acid, methyl ester) and 9,12,15-octadecatrienoic acid, (Z, Z, Z)- (linolenic acid), there are also 6,9,12,15-docosatetraenoic acid, methyl ester and 9-t-butyltricyclo [4.2.1.1(2,5)] decane-9,10-diol which are the main chemical constituents identified in the methanolic extract of *Ficus microcarpa* with peak area percentages of 18.44%, 14.48%, 9.09% and 8.65%, respectively.

**Table 5:** Metabolites identified in the methanolic extract of *Ficus microcarpa* by GC-MS

**No.**	**Rt.**	**Area**	**Compounds**	**Molecular** **Formula**	**Molecular** **weight**
1	7.09	0.73	1,2-dimethyl-Bicyclo [2.2.1] heptan-2-ol	C_9_H_15_	142
2	10.71	1.63	Cyclohexene, 1-(3-ethoxy-1-propenyl)-, (Z)-	C_11_H_18_O	166
3	12.35	0.68	3,5-Heptadienal, 2-ethylidene-6-methyl	C_10_H_14_O	150
4	13.13	0.81	2,6-dimethoxyphenol	C_8_H_10_O_3_	154
5	17.19	0.88	4-Mercaptobenzoic acid, S-methyl-, methyl ester	C_9_H_10_O_2_S	182
6	18.67	0.73	2-Methyl-4-(2,6,6-trimethylcyclohex − 1-enyl) but-2-en-1-ol	C_14_H_24_O	208
7	19.66	0.86	(3,8,8-Trimethyl-1,2,3,4,5,6,7,8-octahydro-2-naphthalenyl) methyl acetate	C_16_H_26_O_2_	250
8	21.32	1.16	Desulphosinigrin	C_10_H_17_NO_6_S	279
9	22.73	3.87	Unidentified	---	---
10	23.05	1.11	Unidentified	---	---
11	25.33	5.11	*α*-Methyl-l-sorboside	C_7_H_14_O_6_	194
12	26.44	5.42	Hexadecanoic acid, methyl ester (Methyl palmitate)	C_17_H_34_O_2_	270
13	27.32	6.14	Palmitic acid	C_16_H_32_O_2_	256
14	27.76	0.48	Palmitic acid, ethyl ester	C_18_H_36_O_2_	284
**15**	**29.54**	**18.44**	**9**,**12**,**15-Octadecatrienoic acid**,** methyl ester**,** (Z**,** Z**,** Z)-** **(Linolenic acid**,** methyl ester)**	**C**_**19**_ **H**_**32**_ **O**_**2**_	**292**
16	29.65	0.30	10-Octadecenoic acid, methyl ester	C_19_H_36_O_2_	296
**17**	**30.46**	**14.48**	**9**,**12**,**15-Octadecatrienoic acid**,** (Z**,** Z**,** Z)-** **(Linolenic acid)**	**C**_**18**_ **H**_**30**_ **O**_**2**_	**278**
18	30.76	0.87	Ethyl 9,12,15-octadecatrienoate (Linolenic acid ethyl ester)	C_20_H_34_O_2_	306
19	32.15	0.38	cis-5,8,11,14,17-Eicosapentaenoic acid	C_20_H_30_O_2_	302
20	32.42	0.73	5,8,11,14-Eicosatetraenoic acid, methyl ester, (all-Z)-	C_21_H_34_O_2_	318
**21**	**32.68**	**8.65**	**9-t-Butyltricyclo [4.2.1.1(2**,**5)] decane-9**,**10-diol**	**C**_**14**_ **H**_**24**_ **O**_**2**_	**224**
22	32.98	0.68	Butyl 6,9,12-hexadecatrienoate	C_20_H_34_O_2_	306
**23**	**33.22**	**9.09**	**6**,**9**,**12**,**15-Docosatetraenoic acid**,** methyl ester**	**C**_**23**_ **H**_**38**_ **O**_**2**_	**346**
	**33.32**				
24	33.60	3.48	6,9,12-Octadecatrienoic acid, methyl ester	C_19_H_32_O_2_	292
25	34.30	0.77	5,8,11,14-Eicosatetraenoic acid, methyl ester, (all-Z)-	C_21_H_34_O_2_	318
26	34.66	0.66	9,12,15-Octadecatrienoic acid, 2,3-dihydroxypropyl ester, (Z, Z, Z)-	C_21_H_36_O_4_	352
Total identified				83.34	

**Table 6:** Name, nature, hit spectrum, chemical structure, and reported bioactivity of the major metabolites in the ME of *F. m*

No.	Name	Nature of compound	Area %	Hit Spectrum	Chemical structure	Biological activity
1.	9,12,15-Octadecatrienoic acid, methyl ester, (Z, Z, Z)-(Linolenic acid, methyl ester)	Fatty acid ester	18.44			Antioxidant, antimicrobial, anti-inflammatory, hepatoprotective, and antimalarial activities [35–38]
2.	9,12,15-Octadecatrienoic acid, (Z, Z, Z)-(Linolenic acid)	Fatty acid	14.48			Analgesic, anti-inflammatory, antioxidant, antipyretic, antimicrobial, anticancer, hypocholesterolemic, hepatoprotective, nematocidal activities [39, 40]
3.	6,9,12,15-Docosatetraenoic acid, methyl ester	Fatty acid ester	9.09			Anticancer and antiatherosclerosis activities [41, 42]
4.	9-t-Butyltricyclo [4.2.1.1(2,5)] decane-9,10-diol	Tricyclic compound	8.65			Anticancer [43]

### Molecular docking study

**Fig. 4:**
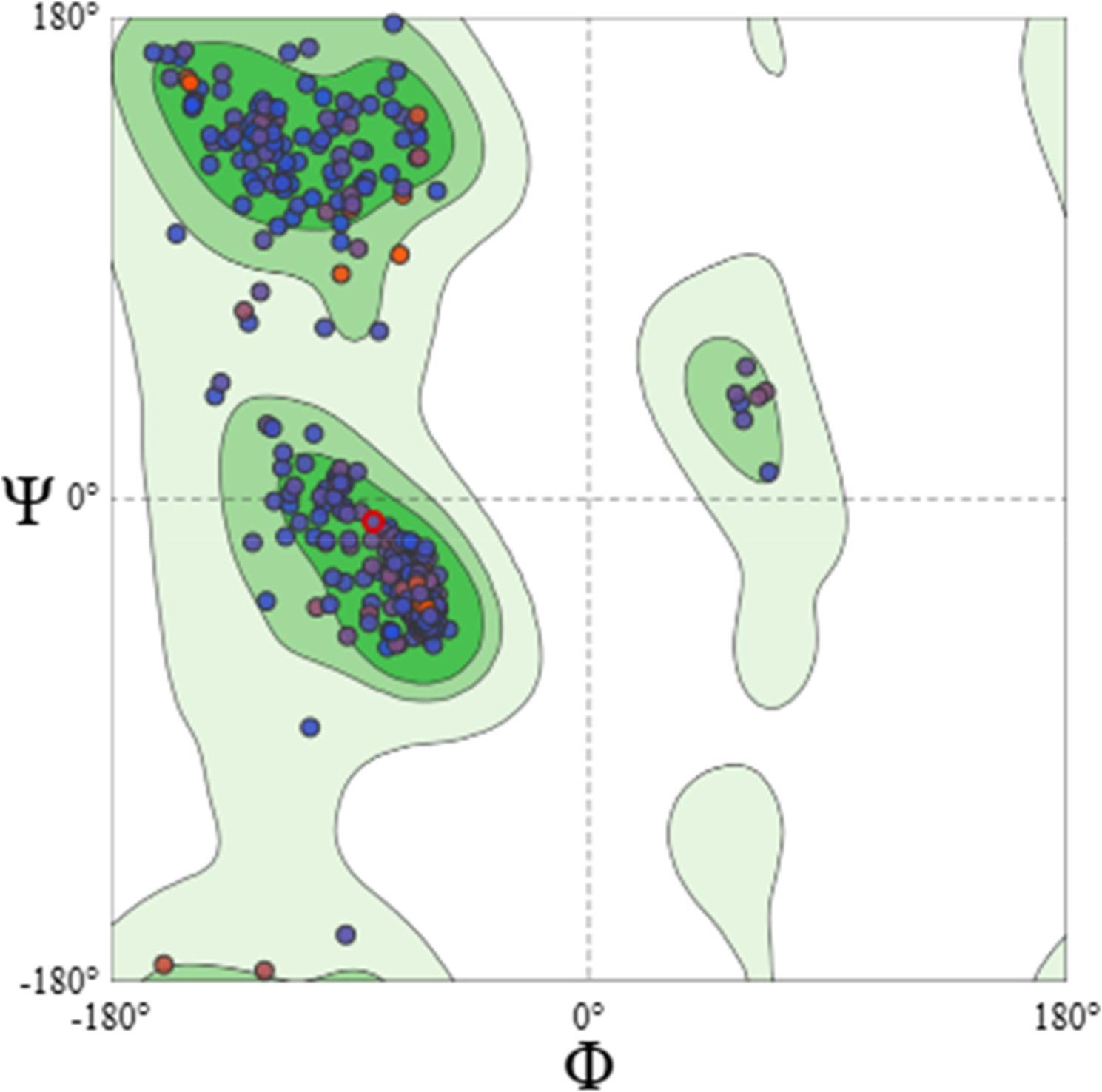
The Ramachandran plot from the MolProbity analysis highlights the amino acids’ preferred and forbidden regions

Protein homology modeling was used to develop the 3D structure of *T. spiralis* tubulin beta chain because it was not available in the PDB database at the time of the work. The model was built and tested for its ability to resemble the natural *T. spiralis* tubulin beta chain. The B-chain of the crystal structure of tubulin-colchicine complex from *Bos taurus* in complex with colchicine inhibitor (PDB ID: 4o2b) was used as the template, which offered high query coverage (95%) and good sequence identity (95.25%) with the target protein. Several parameters were used to assess the models’ quality:

- The GMQE score, ranging from 0 to 1, indicated good structural reliability for the model (0.82 for the mode).
- MolProbity analysis of the Ramachandran plot revealed a high percentage of the model’s preferred residues (98.35%).
- The outliers’ low percentage (0.82%) suggests good model stability. (Fig. 4)

**Fig. 5:**
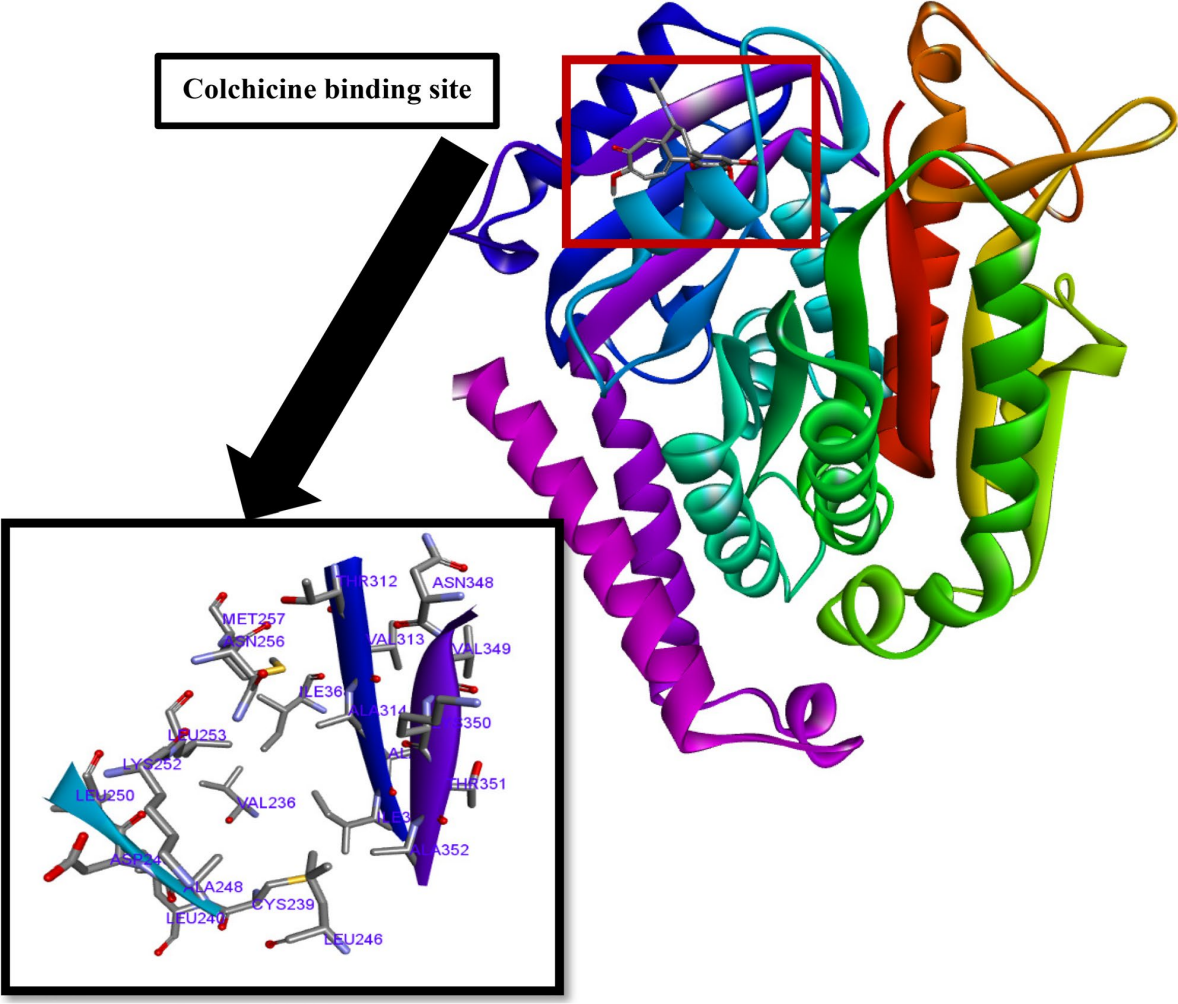
The 3D structure of the constructed model showing the colchicine binding site

The proposed binding site of colchicine is present at the interface of the α/β-subunits within a tubulin heterodimer (in the B monomer N-terminal domain). In β-tubulin models, the proposed binding site comprises several highly conserved hydrophobic amino acids, along with some hydrophilic residues (Fig. 5).

To test the reliability of the docking protocol, colchicine was redocked in the modeled colchicine binding pocket of the *T. spiralis* β-tubulin homology model. The predicted pose showed an RMSD of 0.87 Å with respect to the reference conformation (co-crystallized colchicine extracted from the template PDB: 4O2B). This value is significantly lower than the accepted threshold value of 2.0 Å and confirms that the docking parameters and grid can reliably reproduce the native binding geometry. It also supports the reliability of the docking scores and binding mode predictions for the test compounds.

The results of the molecular docking analysis (Table 7) offer valuable insights into the potential interactions between the tested compounds and the target protein. The binding free energies (measured in kcal/mol) indicate the binding affinity between the tested compounds and the receptor, with lower values indicating a more stable interaction. According to the data, all investigated compounds appear to have a higher binding affinity for the tubulin beta chain than the reference ligand albendazole (-5.895 kcal/mol) but a lower affinity than the co-crystallized ligand colchicine (-8.269 kcal/mol).

**Table 7:** The docking binding free energies

Compounds	ΔG (kcal/mol)
Co-crystallized ligand (colchicine)	**-8.269**
9,12,15-Octadecatrienoic acid, (Z, Z, Z)- (Linolenic acid)	**-6.015**
9,12,15-Octadecatrienoic acid, methyl ester, (Z, Z, Z)- (Linolenic acid, methyl ester)	**-5.952**
6,9,12,15-Docosatetraenoic acid, methyl ester	**-6.399**
9-t-Butyltricyclo [4.2.1.1(2,5)] decane-9,10-diol	**-5.977**
Albendazole	**-5.895**

**Fig. 6:**
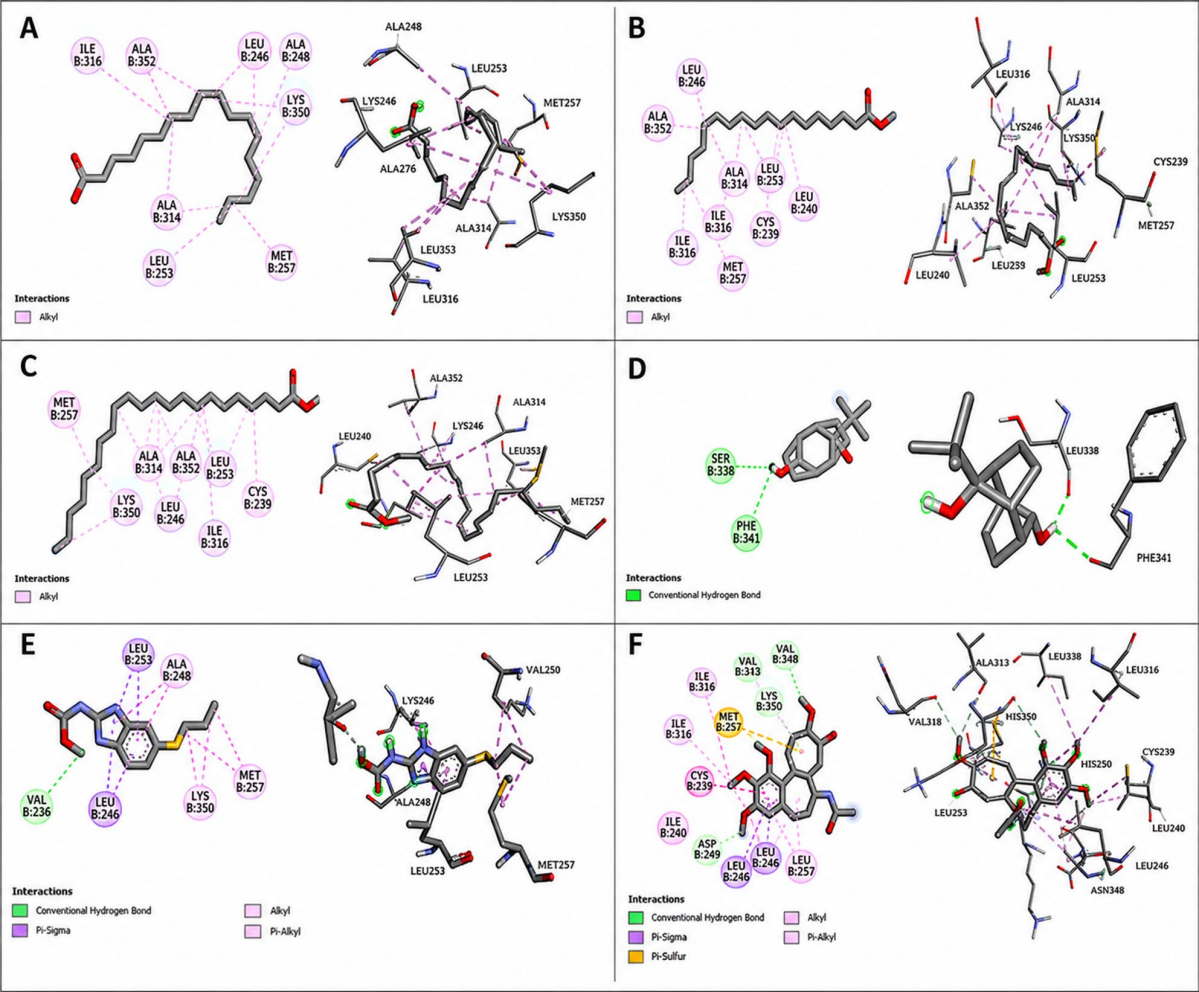
**A** Interactions of tubulin beta chain with linolenic acid, linolenic methyl ester, docosatetraenoic acid, methyl ester, and butyltricyclodecane diol (**A**-**D**, respectively) in addition to two reference compounds, albendazole (**E**) and colchicine (**F**). (3D (right) and 2D (left)

Figure 6 shows that hydrophobic interactions are the key interactions for all tested compounds, except for the 9-t-Butyltricyclo[4.2.1.1(2,5)]decane-9,10-diol ligand, for which hydrogen bonds are the sole interactions.

## Discussion

Trichinosis is a disease that many mammals, including humans, can contract. Public health interventions have substantially improved disease control; however, sporadic outbreaks continue to occur globally [44]. Numerous health benefits, including antioxidant, antimicrobial, antidiabetic, anticancer, antibacterial, and antirheumatic properties, have been demonstrated for the ficus plant. *Ficus* species have been shown in numerous studies to have medicinal promise against some parasites [11].

The plant extracts of seven *Ficus* species were evaluated for their anti-*T. spiralis* effects in vitro. Our parasitological results proved that the survival number of worms and LC_50_ of *Ficus microcarpa* methanolic extract (*F.m* ME) were the lowest, with an LC_50_ of 12.8 µg/mL, followed by its aqueous extract with an LC_50_ of 18.9 µg/mL, compared with the group that received albendazole with an LC_50_ of 52.5 µg/mL. Scanning electron microscopy revealed marked destruction of the adult worms in the albendazole-treated group. Regarding *Ficus*, extracts from *F. microcarpa* showed the most promising effects in in vitro studies, followed by *F. sycomorus* and *F. nitida*. This goes in agreement with Amorin et al. [45], who proved that the latex of *F. insipida*, administered by the intragastric route, was effective as an anti-helminthic therapeutic agent in the removal of oxyurids *Syphacia obvelata*, *Aspiculuris tetraptera*, and the cestode *Vampirolepis nana.* Our results also align with those of Siyadatpanah. et al. [46], whose study showed a marked reduction in the LC_50_ of *Leishmania major* after using *Ficus carica* extracts, which considerably lowered the parasite burden in lesions.

*Ficus* species anti-parasite activity could be attributed to the presence of phenolic compounds, notably the quercetin derivative, and to tannin-rich components, which are more effective against gastrointestinal worms. The antiparasitic efficacy is due to the synergistic effects of substances. Tannin-rich plants have been used to control gastrointestinal parasites by inducing structural changes in the parasites, limiting larval growth, and impairing female fertility and egg integrity [47].

According to the obtained data from GC-MS analysis on the potent, effective methanolic extract (the major bioactive compounds identified include both fatty acid esters and fatty acids, with varying biological activities. The most abundant compound was 9,12,15-Octadecatrienoic acid, methyl ester (methyl linolenate), a fatty acid ester, accounting for 18.44% of the total area. This compound is reported to exhibit antioxidant [35], antimicrobial [36], anti-inflammatory [37], hepatoprotective [38], diuretic [48], and antimalarial [36] properties. The second most prevalent was 9,12,15-Octadecatrienoic acid (linolenic acid), a fatty acid constituting 14.48%, known for its analgesic [39], anti-inflammatory [40], antioxidant [39], antipyretic [40], antimicrobial [39], anticancer [40], hypocholesterolemic [39], hepatoprotective [40], and nematocidal [39] activities. 6,9,12,15-Docosatetraenoic acid, methyl ester, another fatty acid ester with 9.09% area, has been associated with anticancer and antiatherosclerosis effects [41, 42].

Additionally, 9-t-Butyltricyclo[4.2.1.1(2,5)]decane-9,10-diol, a tricyclic compound accounting for 8.65%, was identified; however, its biological activity has not been reported in the current literature. The homology modeling of the *T. spiralis* tubulin beta chain successfully addressed the absence of a crystallized structure in the PDB database (as of June 2025). The high sequence identity (95.25%) and coverage (95%) with the Bos taurus tubulin B-chain (PDB ID: 4O2B) provided a robust template for modeling, consistent with established guidelines that ≥ 50% sequence identity ensures reliable homology predictions. Structural validation further reinforced the model’s reliability: a GMQE score of 0.82 (Swiss-Model) indicates high per-residue quality. At the same time, the Ramachandran plot (98.35% favored regions, 0.82% outliers) aligns with benchmarks for well-resolved crystal structures [49]. Docking analysis revealed that all tested compounds exhibited stronger binding affinity to the tubulin beta chain than albendazole, with 6,9,12,15-docosatetraenoic acid methyl ester showing the highest affinity (-6.399 kcal/mol). While none exceeded colchicine’s binding strength (-8.269 kcal/mol), the scores suggest inhibitory potential comparable to other antiparasitic agents targeting the colchicine site [50]. The predominance of hydrophobic interactions aligns with the conserved nature of this binding pocket, though 9-t-butyltricyclodecane diol’s hydrogen-bonding profile highlights opportunities for selective ligand design [51].

In our study, we chose the colchicine binding site (CBS) as a docking target for several reasons. First, there is extensive experimental evidence that benzimidazole anthelmintics, such as albendazole, competitively inhibit colchicine binding to tubulin and that they share overlapping or adjacent binding sites in the CBS. For instance, studies on *T. spiralis* β-tubulin have shown that benzimidazole-2-carbamate derivatives bind to a pocket that partially overlaps the colchicine-binding site, which contains important residues related to drug resistance, such as F167Y, E198A, and F200Y [52]. Second, CBS is known to bind structurally diverse molecules of different sizes, shapes, and affinities as a promiscuous binding site, making it a relevant target for screening phytochemicals with diverse chemical scaffolds [53]. Third, CBS remains an important pharmacological target for antiparasitic drug development because it inhibits microtubule polymerization, which is essential for parasite survival [54].

However, we know that β-tubulin harbors several pharmacologically distinct ligand-binding sites, e.g., the taxol, vinblastine, and colchicine sites. Moreover, studies have identified alternative or additional interaction sites within the colchicine-binding domain that may be targeted by other chemical classes of anthelmintics, suggesting that some compounds could bind to regions adjacent to or different from the classical colchicine pocket. For example, it has been proposed that triclabendazole binds to a distinct site within the colchicine-binding domain of *Fasciola hepatica* β-tubulin rather than competing directly with colchicine [54]. So, our docking results provide a testable hypothesis for CBS interaction. Still, we cannot exclude that other binding sites or extended regions may also contribute to the anthelmintic activity of the tested extracts.

Overall, the in silico docking analyses indicate that the identified phytocompounds interact with thermodynamically favorable geometries within the colchicine-binding pocket of *T. spiralis* β-tubulin, with binding energies comparable to or better than those of albendazole. These results provide a rationale for considering β-tubulin as a potential molecular target, but several methodological limitations should be acknowledged. Molecular docking uses static receptor models and empirical scoring functions that approximate binding affinity but do not account for protein flexibility, solvent effects, or the kinetic and thermodynamic determinants of ligand binding in vivo [55]. Furthermore, scoring functions are not universal and can vary in accuracy depending on the ligand’s chemical class and the nature of the binding site [56]. Hence, these computational predictions should be considered as hypothesis-generating and used as a basis for mechanistic validation studies. “Further studies should involve tubulin polymerization assays to evaluate functional inhibition, molecular dynamics simulations to assess binding stability over time, and bioassay-guided fractionation to test isolated compounds to establish whether β-tubulin inhibition is indeed the primary mechanism responsible for the observed anthelmintic activity.

Although molecular docking suggested that some phytocompounds from the tested *Ficus* species had higher predicted binding affinities (lower ΔG values) to the target protein than albendazole, the in vitro anthelmintic activity of the crude extracts did not always directly correlate with these in silico predictions. Some extracts, particularly *F.m* ME, were more effective than albendazole, while others needed much higher concentrations to achieve similar worm mortality. This difference is not surprising, as computational binding energy alone does not fully predict in vitro efficacy. Multiple studies have shown that there is no linear correlation between predictive ΔG values and experimental IC_50_ data. Simplified receptor-ligand models and static receptor conformations in computational predictions do not account for complex biological behavior [57]. This observation can be ascribed to several biological and physicochemical factors, including poor membrane permeability, low aqueous solubility, efflux via parasite transporters, and metabolic inactivation by parasite or medium components [58]. Moreover, the uptake of anthelmintic drugs by nematodes is a complex process that involves passive diffusion, facilitated transport through channels, and even endocytosis, which can significantly affect the drug’s accessibility to intracellular targets and is not accounted for in docking simulations [59].

Furthermore, crude extracts are complex mixtures, and synergistic or antagonistic interactions among different phytochemicals can influence their overall bioactivity. In contrast, docking simulations evaluate compound-target interactions of single compounds in isolation [60]. Thus, in silico docking is informative about potential molecular targets and structure–activity relationships, but should be considered a complementary tool rather than a direct indicator of whole-organism efficacy [61].

These findings suggest the potential of the identified natural compounds as possible tubulin-targeting agents against *T. spiralis*.

### Limitations of the current study and future directions

Although computational docking binding energies are good indicators of hypothetical interactions that could explain the mechanism of action of molecules with antimicrobial activity, they cannot replace experimental validation or offer conclusive proof of enzymatic inhibition; additional in vitro enzyme kinetics are needed.

In the current study, we used DMSO (0.5% -1%) as a solvent in the biological assays. Due to the potential for DMSO interference, further in vivo studies are required to confirm these in vitro findings, especially to rule out any solvent-related artifacts.

## Conclusion

This study presented new data supporting the antiparasitic ability of *Ficus* species extracts against *T. spiralis*. Of the extracts tested, *Ficus microcarpa* showed the greatest therapeutic potential, reducing parasite levels by a large margin in vitro. Ultrastructural studies further supported the significant injury inflicted on the parasite in terms of morphology following treatment. The mode of action may stem from various bioactive compounds, especially phenolics and fatty acid derivatives, known for their antioxidant, antimicrobial, and anti-inflammatory properties. Molecular docking studies supported these results, with high binding affinities among the major compounds and the tubulin beta chain of *T. spiralis*, an essential protein for parasite survival. Collectively, these results emphasize the promising potential of *Ficus* species, especially *F. microcarpa*, as natural sources for the development of antiparasitic agents for the therapy of trichinellosis, for further investigation.

## Supplementary Information

Supplementary Material 1.

## Data Availability

The datasets generated or analyzed during the current study are included in the manuscript.

## Abbreviations

AE: Distilled water
ALB: Albendazole
DMSO: Dimethyl sulfoxide
F.: Ficus
GAE: Gallic acid equivalent
GC-MS: Gas chromatography/mass spectrometry
GMQE: General Model Quality Estimate
ME: MeOH
mg: Milligram
QMEAN: Qualitative Model Energy Analysis
spp.: species
SEM: Scanning electron microscopy
TPC: Total Phenolic Content
T. spiralis: Trichinella spiralis
