# Supplementary material for: Chemical investigation and anti-trichinellosis activity of some ficus species leaves: in silico supported in vitro study
Source: BMC Complement Med Ther. 2026 Aug 14;26:246. doi: 10.1186/s12906-026-05500-5 (PMC13474506; doi:10.1186/s12906-026-05500-5)
Supplement: Supplementary file 1 [file 12906_2026_5500_MOESM1_ESM.docx]

**Table 1. Survival number of *T. spiralis* adults and** **LC50 of different *Ficus* species**

| **Survival number ( of adult worms** | | | | | | | | |
| --- | --- | --- | --- | --- | --- | --- | --- | --- |
| **Drugs** | **Dose µg/ml** | **Exposure times (h)** | | | | | **ANOVA**  ***p*** | **LC_50_ (48 h)** |
|  |  | **1** | **4** | **24** | **48** | **72** |  |  |
| **Parasite control** | **-----** | 24.5±0.71 | 24±1.41 | 22.5±0.71 | 19.5±0.7 | 17.5±0.7 | **-----** | **-----** |
| **DMSO control** | **-----** | 24.5±0.71 | 24±00 | 22±1.4 | 19.5±0.7 | 17.5±0.71 | **-----** | **-----** |
| **ALB** | **400** | 3.5±0.71** | 0.5±0.707** | 0 | 0 | 0 | 0.002 | **52.5 µg/ml** |
|  | **200** | 11.5±0.71* | 8±1.414*** | 0.5±0.707*** | 0 | 0 | <0.0001 |  |
|  | **100** | 19.5±0.71** | 15.5±0.707*** | 7.5±0.707*** | 0 | 0 | <0.0001 |  |
|  | **50** | 22.5±0.707*** | 20.5±0.707 | 18 | 12.5±0.707 | 9 | 0.0421 |  |
|  | **25** | 23.5±0.707 | 22±1.414 | 20±1.414 | 16.5±2.121 | 14±1.414* | 0.185 |  |
| ***p*-value:** | | <0.0001 | <0.0001 | 0.0021 | 0.0248 | 0.0281 |  | |
| ***F.b* AE** | **400** | 19.5±0.707 | 16.5±0.707* | 14.5±0.707** | 11.5±0.71*** | 7±1.414* | 0.0002 | **302.17 µg/ml** |
|  | **200** | 20±2.83 | 17.5±0.71 | 15.5±0.71* | 12±1.414** | 10** | 0.006 |  |
|  | **100** | 24*** | 23*** | 20*** | 18*** | 12*** | <0.0001 |  |
|  | **50** | 24*** | 21*** | 20*** | 18*** | 16*** | <0.0001 |  |
|  | **25** | 25*** | 25*** | 23*** | 21*** | 19*** |  |  |
| **p-value:** | | 0.0022 | 0.0015 | 0.0004 | 0.0008 | 0.0003 |  | |
| ***F.b* ME** | **400** | 15±1.414 | 13.5±2.12 | 11±1.414 | 9.5±3.5 | 4±1.414 | 0.02 | **291.9****9 µg/ml** |
|  | **200** | 17.5±2.12 | 16±1.414 | 13.5±0.7 | 12±2.8 | 6.5±0.7 | 0.009 |  |
|  | **100** | 23.5±0.707 | 22.5±0.71 | 20 | 19±1.4 | 10.5±0.707 | <0.0001 |  |
|  | **50** | 24 | 20 | 17.5±0.707 | 16.5±0.71 | 12.5±0.71 | <0.0001 |  |
|  | **25** | 25 | 24.5 | 21.5±0.71 | 20.5±0.707 | 17.5±0.7 | 0.0004 |  |
| **p-value:** | | <0.0001 | 0.0007 | 0.0004 | 0.0018 | 0.0005 |  | |
| ***F.c* AE** | **400** | 11±2.12 | 8.5±1.414 | 5±1.414*** | 0.5±0.7 | 0 | 0.26 | **86.71** **µg/ml** |
|  | **200** | 17.5±1.41 | 16±2.12 | 8.5±2.12 | 2.5±2.12 | 0 | 0.41 |  |
|  | **100** | 20.5±0.7*** | 19.5±0.71*** | 16 | 7.5±0.707*** | 0.5±0.71*** | <0.0001 |  |
|  | **50** | 22*** | 21*** | 19*** | 17*** | 15*** | <0.0001 |  |
|  | **25** | 25*** | 24*** | 21*** | 19*** | 16*** | 0.0004 |  |
| **p-value:** | | <0.0001 | <0.0001 | <0.0001 | 0.0013 | 0.0194 |  | |
| ***F.c* ME** | **400** | 9.5±0.7*** | 8±1.41*** | 3.5±0.7*** | 0 | 0 | 0.32 | **65.76 µg/ml** |
|  | **200** | 14.5±0.7 | 10.5±0.7 | 3*** | 0 | 0 | 0.003 |  |
|  | **100** | 19.5±0.7 | 18.5±0.707 | 15 | 4.5±2.12 | 0 | <0.0001 |  |
|  | **50** | 21±1.41 | 19.5±0.71 | 16.5±0.7 | 14.5±0.7 | 10.5±0.7* | 0.0005 |  |
|  | **25** | 24.5±0.7*** | 22.5±0.7 | 18.5±0.7 | 16±1.41** | 14±1.41 | 0.0008 |  |
| **p-value:** | | <0.0001 | <0.0001 | 0.0010 | 0.0048 | 0.0242 |  | |
| ***F.e* AE** | **400** | 16±1.41 | 15 | 10.5±0.707*** | 6.5±0.707*** | 2*** | <0.0001 | **213.33 µg/ml** |
|  | **200** | 18±1.41 | 17 | 13.5±0.707 | 10.5±0.707 | 7.5±0.707 | <0.0001 |  |
|  | **100** | 23.5±0.7 | 22.5±0.707 | 19.5±0.7 | 16.5±0.707 | 11±1.41 | 0.0003 |  |
|  | **50** | 24 | 21 | 20 | 18 | 16 | 0.0066 |  |
|  | **25** | 25 | 24±1.41 | 21.5±0.71 | 19.5±0.707 | 16±1.41 | 0.0014 |  |
| **p-value:** | | <0.0001 | 0.0003 | <0.0001 | <0.0001 | <0.0001 |  | |
| ***F.e* ME** | **400** | 14±1.41 | 13±1.41 | 7.5±0.707 | 2.5±0.707 | 0 | 0.0001 | **163.3****5 µg/ml** |
|  | **200** | 16 | 15.5±0.707 | 11±1.414 | 7±1.414 | 3±1.414 | 0.0004 |  |
|  | **100** | 22.5±0.707 | 20.5±0.707 | 17.5±0.707 | 16±1.414 | 9.5±0.707 | 0.0002 |  |
|  | **50** | 23 | 20 | 18 | 15 | 13 | 0.05 |  |
|  | **25** | 24.5±0.707 | 22.5±2.12 | 19.5±0.707 | 17.5±0.707 | 15.5±2.12 | 0.008 |  |
| **p-value:** | | <0.0001 | 0.0001 | <0.0001 | <0.0001 | <0.0001 |  |  |
| ***F.m* AE** | **400** | 3.5±0.707* | 1.5±0.707* | 0 | 0 | 0 | 0.0019 | **18.859** **µg/ml** |
|  | **200** | 6.5±0.707*** | 3*** | 0 | 0 | 0 | <0.0001 |  |
|  | **100** | 9.5±0.707*** | 6±1.414*** | 2±1.414 | 0 | 0 | 0.0006 |  |
|  | **50** | 15* | 13*** | 10*** | 6** | 3.5±0.707*** | <0.0001 |  |
|  | **25** | 19** | 16±1.414*** | 11*** | 8*** | 5*** | <0.0001 |  |
| **p-value:** | | 0.0006 | 0.0022 | 0.0085 | 0.0235 | 0.0257 |  |  |
| ***F.m* ME** | **400** | 2.5±0.71 | 0 | 0 | 0 | 0 | 0.1850 | **12.796 µg/ml** |
|  | **200** | 4.5±0.71 | 0 | 0 | 0 | 0 | 0.1188 |  |
|  | **100** | 9 | 4.5±0.71* | 1 | 0 | 0 | 0.0779 |  |
|  | **50** | 13±1.41 | 9.5±0.707 | 7.5±0.707 | 4.5±0.707 | 2 | 0.0359 |  |
|  | **25** | 18±1.41*** | 15±1.414 | 9.5±0.707 | 6.5±0.707 | 3.5±0.7 | 0.0193 |  |
| **p-value:** | | 0.0009 | 0.0048 | 0.0131 | 0.0261 | 0.0324 |  | |
| ***F.n* AE** | **400** | 5 | 4 | 1 | 0 | 0 | <0.0001 | **53.59 µg/ml** |
|  | **200** | 12 | 10 | 4 | 0 | 0 | <0.0001 |  |
|  | **100** | 17 | 15 | 10 | 3 | 0 | <0.0001 |  |
|  | **50** | 20 | 17 | 16 | 12 | 10 | <0.0001 |  |
|  | **25** | 23 | 20 | 18 | 15 | 11 | <0.0001 |  |
| **p-value:** | | <0.0001 | <0.0001 | 0.0001 | 0.0070 | 0.0226 |  |  |
| ***F.n* ME** | **400** | 5 | 3 | 1 | 0 | 0 | <0.0001 | **42.87 µg/ml** |
|  | **200** | 11 | 8 | 0 | 0 | 0 | <0.0001 |  |
|  | **100** | 15 | 13 | 7 | 1 | 0 | <0.0001 |  |
|  | **50** | 18 | 15 | 14 | 9 | 7 | <0.0001 |  |
|  | **25** | 22 | 19 | 16 | 14 | 7 | <0.0001 |  |
| **p-value:** | | <0.0001 | <0.0001 | 0.0030 | 0.0177 | 0.0232 |  |  |
| ***F.s* AE** | **400** | 5 | 4 | 0 | 0 | 0 | <0.0001 | **45.196 µg/ml** |
|  | **200** | 11 | 8 | 2 | 0 | 0 | <0.0001 |  |
|  | **100** | 13 | 11 | 6 | 0 | 0 | <0.0001 |  |
|  | **50** | 19 | 15 | 13 | 10 | 7 | <0.0001 |  |
|  | **25** | 22 | 19 | 17 | 15 | 10 | <0.0001 |  |
| **p-value:** | | <0.0001 | 0.0001 | 0.0012 | 0.0255 | 0.0246 |  |  |
| ***F.s* ME** | **400** | 4 | 3 | 0 | 0 | 0 | <0.0001 | **31.46** **µg/ml** |
|  | **200** | 9 | 6 | 0 | 0 | 0 | <0.0001 |  |
|  | **100** | 12 | 9 | 5 | 0 | 0 | <0.0001 |  |
|  | **50** | 16 | 12 | 10 | 7 | 4 | <0.0001 |  |
|  | **25** | 20 | 17 | 14 | 11 | 8 | <0.0001 |  |
| **p-value:** | | <0.0001 | 0.0004 | 0.0027 | 0.0266 | 0.0344 |  | |
| ***F.v* AE** | **400** | 9 | 5 | 2 | 0 | 0 | <0.0001 | **63.196 µg/ml** |
|  | **200** | 15 | 13 | 5 | 0 | 0 | <0.0001 |  |
|  | **100** | 19 | 17 | 12 | 4 | 0 | <0.0001 |  |
|  | **50** | 23 | 20 | 18 | 14 | 11 | <0.0001 |  |
|  | **25** | 25 | 24 | 20 | 17 | 13 | <0.0001 |  |
| **p-value:** | | <0.0001 | <0.0001 | 0.0001 | 0.0055 | 0.0226 |  | |
| ***F.v* ME** | **400** | 8±1.41* | 4±1.41** | 0.5±0.707** | 0 | 0 | 0.0014 | **49.81µg/ml** |
|  | **200** | 14±1.41* | 10*** | 2.5±0.71* | 0 | 0 | <0.0001 |  |
|  | **100** | 18.5±1.41 | 15.5±2.12* | 10±1.41*** | 0.5±0.7*** | 0 | 0.0002 |  |
|  | **50** | 21.5±2.12 | 19±4.243 | 17±2.83 | 12.5±3.54 | 8.5±2.12* | 0.0418 |  |
|  | **25** | 23±1.41 | 22±1.41 | 18.5±3.54 | 15±4.2 | 13±5.66 | 0.1434 |  |
| ***p*-value:** | | <0.0001 | <0.0001 | 0.0007 | 0.0254 | 0.0397 |  | |
